# A Novel Infection Protocol in Zebrafish Embryo to Assess *Pseudomonas aeruginosa* Virulence and Validate Efficacy of a Quorum Sensing Inhibitor In Vivo

**DOI:** 10.3390/pathogens10040401

**Published:** 2021-03-29

**Authors:** Pauline Nogaret, Fatima El Garah, Anne-Béatrice Blanc-Potard

**Affiliations:** 1 Laboratory of Pathogen-Host Interactions (LPHI), Université Montpellier, CEDEX 05, 34095 Montpellier, France; pauline.nogaret@umontpellier.fr; 2 CNRS, UMR5235, CEDEX 05, 34095 Montpellier, France; 3 Laboratoire de Génie Chimique, Université de Toulouse, CNRS, INPT, UPS, 31432 Toulouse, France; fatima.el-garah@univ-tlse3.fr

**Keywords:** *Pseudomonas aeruginosa*, zebrafish, quorum sensing

## Abstract

The opportunistic human pathogen *Pseudomonas aeruginosa* is responsible for a variety of acute infections and is a major cause of mortality in chronically infected cystic fibrosis patients. Due to increased resistance to antibiotics, new therapeutic strategies against *P. aeruginosa* are urgently needed. In this context, we aimed to develop a simple vertebrate animal model to rapidly assess in vivo drug efficacy against *P. aeruginosa*. Zebrafish are increasingly considered for modeling human infections caused by bacterial pathogens, which are commonly microinjected in embryos. In the present study, we established a novel protocol for zebrafish infection by *P. aeruginosa* based on bath immersion in 96-well plates of tail-injured embryos. The immersion method, followed by a 48-hour survey of embryo viability, was first validated to assess the virulence of *P. aeruginosa* wild-type PAO1 and a known attenuated mutant. We then validated its relevance for antipseudomonal drug testing by first using a clinically used antibiotic, ciprofloxacin. Secondly, we used a novel quorum sensing (QS) inhibitory molecule, *N*-(2-pyrimidyl)butanamide (C11), the activity of which had been validated in vitro but not previously tested in any animal model. A significant protective effect of C11 was observed on infected embryos, supporting the ability of C11 to attenuate in vivo *P. aeruginosa* pathogenicity. In conclusion, we present here a new and reliable method to compare the virulence of *P. aeruginosa* strains in vivo and to rapidly assess the efficacy of clinically relevant drugs against *P. aeruginosa*, including new antivirulence compounds.

## 1. Introduction

The environmental bacterium and opportunistic human pathogen *Pseudomonas aeruginosa* is responsible for a variety of acute infections and is a major cause of mortality in chronically infected cystic fibrosis (CF) patients. Due to increased resistance to antibiotics, *P. aeruginosa* has been listed by the World Health Organization (WHO) among pathogenic bacteria for which new antibiotics or alternative therapeutic strategies are urgently needed [1]. Antivirulence strategies have emerged as attractive novel therapeutic approaches that would apply less selective pressure to develop resistance and better preserve microbiota than traditional antimicrobial therapy [2,3]. 

Quorum sensing (QS) allows bacteria to communicate with one another by responding to the population-dependent concentration of small molecules known as autoinducers [4]. *P. aeruginosa* secretes two main classes of autoinducer: acyl-homoserine lactones (HSLs) and several alkylquinolones (AQs), the major one being 2-heptyl-3-hydroxy-4-quinolone (PQS). *P. aeruginosa* harbors three main QS systems (*las*, *rhl*, and *pqs*) that control potent virulence factors [5] and are essential for *P. aeruginosa* pathogenesis, as demonstrated by a study in which *P. aeruginosa* mutants that lacked QS genes caused less lung pathology during acute infection in mice [6]. In addition, sputum cultures from CF patients infected with chronic *P. aeruginosa* contain significant amounts of HSLs and PQS [7]. Thus, QS systems of *P. aeruginosa* have been proposed as potential antivirulence targets [8,9]. In this context, a promising strategy for curtailing *P. aeruginosa* virulence based on a structural analog of C_4_-HSL autoinducer has been identified [10,11]. This compound, named C11 (*N*-(2-pyrimidyl)butanamide), downregulates the *las* and *rhl* QS systems and notably reduces biofilm formation, without affecting *P. aeruginosa* planktonic growth [10]. Moreover, a synergistic antibiofilm activity was found between C11 and the clinically used antibiotics ciprofloxacin, tobramycin, and colistin [10]. Thus, C11 appears as a promising antivirulence molecule that is suitable for in vivo studies because it is stable, not cytotoxic to human cells, and synthetically accessible.

In the context of validation of novel compounds against *P. aeruginosa*, the development of low-cost and rapid animal models is important to assess in vivo efficacy. The zebrafish (*Danio rerio*) has been now widely used as a model for studying host–pathogen interactions [12,13] and is increasingly considered for modeling human infections, including lung infections caused by bacterial pathogens [14,15]. The zebrafish model has a number of advantages over mammalians models of infection in terms of methodological, financial, and ethical issues. Zebrafish are vertebrates, which are genetically and physically closer to humans than invertebrate models. In embryos, only the innate immune system is functional [16], and the optical transparency of the embryonic stages allows the analysis of bacterial infections in real time using fluorescent microorganisms. In addition to its significance as an infection model, the zebrafish embryo is also suitable for in vivo chemical screening [17,18], with the advantage that permeability of the larvae allows the entry of small compounds added directly to the fish water. This model, which also allows drug toxicity to be addressed [19], has been successfully used for drug testing in the context of cancer and infectious diseases [20,21].

The zebrafish embryo model has been used to follow *P. aeruginosa* infections and assess *P. aeruginosa* virulence [22,23,24,25]. *P. aeruginosa* infections are usually established by microinjecting the bacteria into the bloodstream (injection in caudal vein or duct of Cuvier) of 1 or 2 days post-fertilization (dpf) old embryos. This induces an acute infection and mortality of embryos when the amount of bacteria injected exceeds the phagocytic capacity of the embryo. *P. aeruginosa* mutants deficient in QS (*lasR* and *mvfR*) are attenuated in zebrafish embryos infected at 2 dpf [23], supporting that the zebrafish embryo is a suitable infection model to test the efficacy of specific inhibitors targeting QS. 

In the present study, we first aimed to develop a simple and reliable model for *P. aeruginosa* infection in zebrafish embryos based on bath immersion, thus avoiding the time-consuming microinjection step. This new protocol, which is carried out with injured embryos, was first validated to assess *P. aeruginosa* virulence. We next validated its reliability for drug testing using a clinically used antibiotic and the promising C11 QS inhibitory molecule.

## 2. Results

### 2.1. A Bath Infection Model with Injured Zebrafish Embryos Allows the Evaluation of P. aeruginosa Virulence

**Figure 1:**
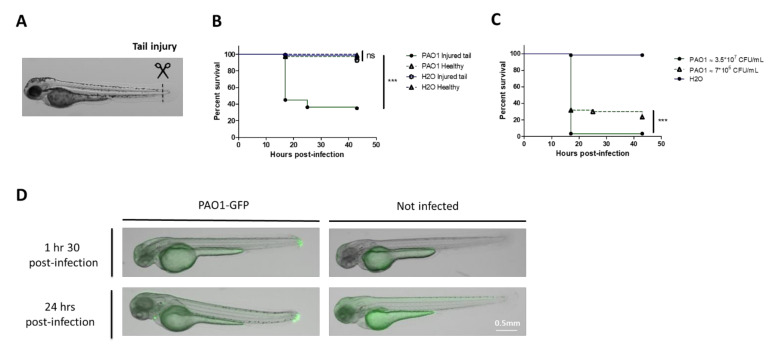
Development of bath infection model with injured zebrafish embryos to assess *P. aeruginosa* virulence. (**A**) Larvae were injured in the tail fin at 48 hours post-fertilization (hpf). (**B**) Survival curves (Kaplan–Meier representation) of healthy or injured embryos (GAB line) immersed at 48 hpf with PAO1 wild-type strain at approximately 2*10^7^ CFU/mL grown in exponential phase or “fish water” (negative control). (**C**) Survival curves (Kaplan–Meier representation) of injured embryos (GAB line) immersed at 48 hpf with PAO1 strain at two different concentrations (3.5 × 10^7^ or 7 × 10^6^ CFU/mL) or fish water (negative control). Results are expressed as the percentage of surviving embryos. Pools of three biologically independent replicates are shown for each survival curve (in each experiment 20 embryos were used per strain). Significant difference at ** *p* < 0.01, *** *p* < 0.001 or no significant difference: ns. (**D**) In vivo imaging of injured embryos immersed with fluorescent *P. aeruginosa* PAO1*-*GFP for 1.5 or 24 hours (left panels). Images represent DIC channel merge to green fluorescence protein (GFP) channel. Scale bar: 0.5 mm. Images of injured embryos immersed with N-phenylthiourea (PTU) without bacteria (right panels) are shown as control. Images are a representative result of three embryos in each condition.

*P. aeruginosa* infection in zebrafish embryos is usually established through microinjection of the bacteria into the embryo, which requires specific expertise and is time-consuming. Our first goal was to set up a reproducible infection assay based on the immersion of embryos with bacteria to bypass the microinjection step. We also aimed to set up a test in 96-well plates to minimize the bath volume in further drug assays. A previous study reported mortality of embryos after bath immersion with PAO1 strain [26], which is, however, not supported by other reports [23,27]. Bath immersion was first performed on healthy embryos at 2 days post-fertilization (dpf) [28], a developmental stage where the mouth is not yet open. We used different bacterial concentrations of a PAO1 strain with two embryos per well in 96-well plates. Viability of embryos was followed for 24 hours but no mortality was observed (Figure S1). The same experiment was then carried out with embryos injured in the tail fin (Figure 1A), because such injury has been shown to provide a portal of entry for bacteria [29]. In contrast to healthy embryos, mortality of injured embryos was observed after 18 hours post-infection (hpi) (Figure 1B), in a bacterial dose-dependent manner (Figure 1C). Imaging embryos shortly after infection revealed the presence of bacteria in the tail 1.5 hours post-infection, which can persist in some live embryos 24 hours after bathing (Figure 1D). Thus, the bath infection model with embryos wounded in the tail appeared a suitable novel mode of infection by *P. aeruginosa*. A deeper microscopy analysis at additional time points will be required for a better understanding of the pathogenesis process. While the image in Figure 1D for time 1.5 hours is representative of the majority of embryos, the image of an alive embryo at time 24 hours is not representative of the diversity of patterns (some embryos cleared the infection and some embryos harbored bacteria that had migrated away from the injury site). We also noticed some variability in the embryo susceptibility to infection, which may depend on various factors, including the age of adult fish. In further experiments, we adjusted the bacterial concentration to achieve a mortality of 60 to 80% of embryos upon infection with PAO1.

**Figure 2:**
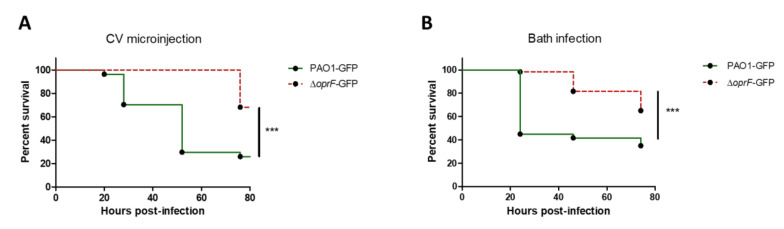
Validation of bath infection model using an attenuated strain of *P. aeruginosa*. (**A**) Kaplan–Meier representation of survival after microinjection of approximately 3500 CFUs of wild-type or Δ*oprF* GFP-expressing *P. aeruginosa* into the caudal vein (CV) of embryos. Pools of three biologically independent replicates are shown (in each experiment 20 embryos were used per strain). (**B**) Kaplan–Meier representation of survival after bath infection of injured embryos with wild-type or Δ*oprF* GFP-expressing *P. aeruginosa* at approximately 10^8^ CFU/mL. Pools of three biologically independent replicates are shown for each survival curve (in each experiment 20 embryos were used per strain).

OprF is a major outer membrane porin involved in maintenance of cell structure, outer membrane permeability, environmental sensing, adhesion, biofilm formation, and virulence [30,31]. To validate the relevance of the new infection model for the assessment of *P. aeruginosa* virulence, we performed a test with wild-type PAO1 and an *oprF* mutant strain, which was previously shown to be attenuated in zebrafish embryos when bacteria are microinjected in the caudal vein [32]. The attenuation profile of the *oprF* mutant in comparison with wild-type PAO1 is similar upon classical microinjection of the bacteria in the caudal vein (Figure 2A) or upon bath infection of injured embryos (Figure 2B), supporting the reliability of the bath model in assessing *P. aeruginosa* virulence.

### 2.2. Validation of the Bath Infection Model to Test Efficacy of a Known Antibiotic

**Figure 3:**
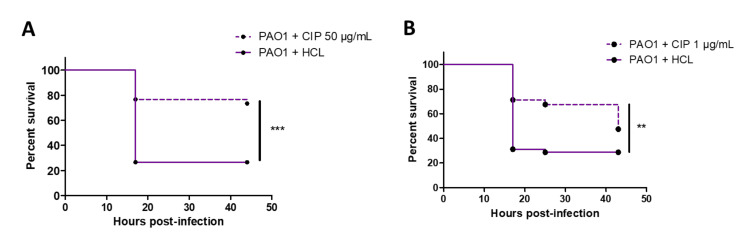
Validation of bath infection model with a known antibiotic. (**A**) Kaplan–Meier representation of the survival of injured zebrafish embryos bath infected with PAO1 strain at approximately 10^8^ CFU/mL and treated with ciprofloxacin 50 µg/mL or hydrochloric acid 0.2 mM (negative control) 2 hours after infection. (**B**) Kaplan–Meier representation of the survival of injured zebrafish embryos bath infected with PAO1 strain at approximately 5 × 10^7^ CFU/mL and treated with ciprofloxacin 1 µg/mL or hydrochloric acid 0.5 mM (negative control) 2 hours after infection. Pools of three biologically independent replicates (with 20 embryos) are shown for each survival curve. *** *p* < 0.001; ** *p* < 0.01.

We next addressed the relevance of the bath infection model to test drug efficacy using ciprofloxacin, a known antipseudomonal antibiotic. Ciprofloxacin added directly in the bath at 50 μg/mL was previously shown to rescue 75% of embryos from lethal infection following microinjection at 50 hours post-fertilization (hpf) with *P. aeruginosa* PA14 strain [23]. In our model, ciprofloxacin was added 2 hours after injury and bacterial immersion, to allow the wound closing and avoid a direct killing of extracellular bacteria in the bath. Ciprofloxacin at 50 µg/mL had a clear and significant protective effect compared to nontreated embryos (Figure 3A). A significant protective effect, of lower amplitude, was also shown at a lower dose of 1 µg/mL (Figure 3B), which corresponds to 10-fold minimum inhibitory concentration (MIC) [33]. Bath infection of injured embryos with *P. aeruginosa* is thus suitable to evaluate the efficacy of antibacterial drugs.

### 2.3. Test of Antivirulence Properties of C11, a Novel Anti-Pseudomonas Molecule Targeting QS

**Figure 4:**
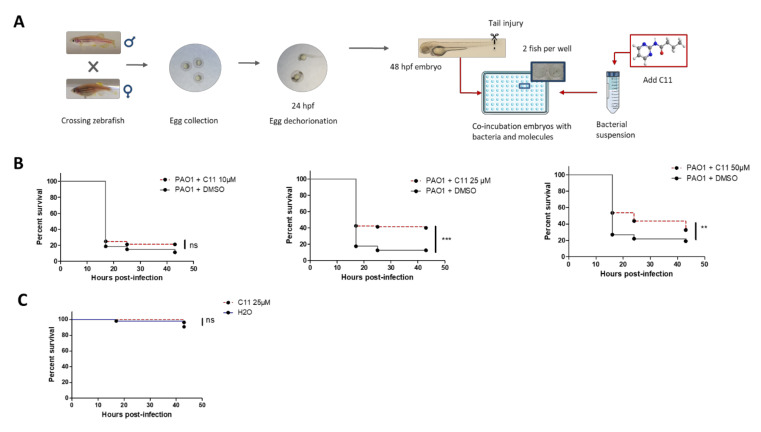
Use of bath infection model to validate novel anti-*Pseudomonas* molecules such as C11 molecule (targeting quorum sensing). (**A**) Schematic protocol for testing antivirulence molecules in embryos bath infected with *P. aeruginosa*. Antivirulence molecules, which do not reduce bacterial viability outside the host, are added together with bacteria. (**B**) The antivirulence efficacy of C11 (dissolved in DMSO) was tested with embryos injured in the tail fin and bath infected with PAO1 suspension at approximately 10^7^ CFU/mL in presence of C11 at 10, 25, or 50 µM. Injured embryos in the control group were treated with PAO1 suspension in presence of DMSO at 0.05, 0.13, or 0.25% (reflecting the amount of DMSO in the C11-treated groups). A significant difference (** *p* < 0.05, *** *p* < 0.001) is found in the survival curve of C11-treated embryos compared to non-treated embryos at 25 or 50 µM. (**C**) The toxicity of C11 at 25 µM was monitored after immersion of embryos injured in the tail fin. For all experiments, the embryo survival was monitored for 45 hours and survival curves were represented with a Kaplan–Meier representation. Graphs represent the pool of three independent experiments (n = 60 larvae in total per condition).

We next evaluated the ability of the protocol of bath infection of injured embryos to test an antivirulence compound in vivo. C11, an antagonistic analog of C_4_-HSL, is a novel molecule targeting *P. aeruginosa* QS [10] that had not previously been evaluated in an animal model. The effect of C11, dissolved in dimethylsulfoxide (DMSO), was tested at different concentrations (10, 25, and 50 µM) on injured embryos in bath immersion with bacterial inoculum at about 10^7^ CFU/mL. C11 has been previously shown to lack classical antibacterial activity and was, therefore, added simultaneously with the bacterial cells following the protocol schematized in Figure 4A. In control embryos immersed with PAO1, DMSO was added to reflect the amount of DMSO in C11-treated embryos (0.05, 0.13, or 0.25%). The C11 compound exhibits a protective effect since a significant reduction in embryo mortality was observed at 25 and 50 µM (Figure 4B). This protective effect was not observed at 10 µM (Figure 4B), thus demonstrating a dose-dependent effect. After this first round of experiments with C11 dissolved in DMSO, we also checked its efficiency upon dilution in water, as previously used [10]. C11 diluted in water at 25 µM was significantly protective (Figure S2), to a similar extent to the effect observed upon dilution in DMSO, thus supporting that DMSO does not contribute to C11 activity in vivo. Importantly, the addition of 25 µM C11 (dissolved in DMSO or H_2_O) was not associated with toxicity for embryos, as shown by the lack of mortality upon addition of the sole molecule (Figure 4C and Figure S2).

## 3. Discussion

The use of the zebrafish embryo as in vivo model for human diseases offers a great opportunity to conduct chemical screens for small molecules in a whole-organism model [34]. The relevance of this model for testing novel therapeutics against *P. aeruginosa* is supported by a recent study using phages [35]. In the present work, we used zebrafish larvae infected by *P. aeruginosa* to test antivirulence molecules for the first time. We firstly developed a novel protocol for infection of zebrafish embryos to assess *P. aeruginosa* virulence and secondly demonstrated its relevance in evaluating the efficacy of antipseudomonal molecules.

*P. aeruginosa* infection in zebrafish embryos is most often established through microinjection of bacterial cells into the embryo. In the context of in vivo drug testing, our aim was first to develop an immersion protocol with *P. aeruginosa* to avoid the microinjection step. A lethal infection after bath immersion of 3 dpf healthy embryos with *P. aeruginosa* was previously reported [26]. However, an associated proteomic analysis indicated induction of hypoxia response in zebrafish embryos exposed to the immersion method, suggesting that healthy larvae suffered from a lack of oxygen when exposed to *P. aeruginosa* by static immersion. Moreover, the host response was also likely corresponding to a response against bacteria that are outside the fish or in contact with its skin [26]. In the present study, we did not observe lethality when healthy embryos at 2 dpf (a developmental stage where the mouth is not yet open) were immersed with PAO1 bacteria, which is in agreement with other reports [23,27]. However, we observed bacterial-dose-dependent mortality when 2 dpf embryos were injured in the tail fin before static immersion with *P. aeruginosa*. Fin amputation in embryos has been widely used as a model of “sterile” wounding injury and inflammation assay [36]. The inflammatory response with the arrival of neutrophils and macrophages after an injury has been well documented, and the embryonic zebrafish fin is also highly regenerative (the tail has been reported to heal within 1 hour after the cut) [37]. Moreover, the use of tail-injured embryos has been shown to increase the susceptibility to infection when compared to 4 dpf healthy larvae by providing an alternative entry gate for the bacterium *Aeromonas hydrophila* [29]. We visualized fluorescent *P. aeruginosa* bacteria within embryos shortly after immersion, which can persist 24 hours after injury in surviving embryos. Thus, bath immersion of tail-injured embryos appears to be a suitable novel protocol to infect zebrafish embryos with *P. aeruginosa*. *P. aeruginosa* OprF was previously reported to play a role in resistance to macrophage clearance during acute infection in zebrafish embryos [32]. To ascertain the pertinence of the new infection mode, we have shown that an *oprF* mutant strain, which is attenuated upon microinjection [32], is similarly attenuated using bath immersion of tail-injured embryos. Further studies, implying deeper microscopy analysis of wild-type and mutant strains, as well as monitoring innate immune response, will be required to decipher the pathogenesis mechanism associated with this novel infection route.

The relevance of tail-injured embryos in assessing the efficacy of drugs against *P. aeruginosa* was first validated with the clinically used antibiotic ciprofloxacin. Protection of embryos from mortality caused by infection was observed at 50 µg/mL, a concentration also previously found to be efficient in protecting embryos microinjected with *P. aeruginosa* [23]. Moreover, we showed that a dose as low as 1 µg/mL was sufficient to significantly protect embryos. After validation of the method with a known antibiotic, we tested a structural analog of C_4_-HSL, the C11 molecule. C11 is a novel inhibitor of *P. aeruginosa* QS [10], and its activity in an animal model was evaluated for the first time in the present study. The addition of C11 reduced embryo mortality, with a significant protective effect at 50 and 25 µM. Thus, our in vivo results confirmed the anti-infective potential of this novel anti-QS compound, without exhibiting any toxicity. Further studies will be required to better understand the mechanism by which C11 increases the defensive ability of zebrafish embryos against *P. aeruginosa* in vivo. In vitro, C11 was shown to inhibit *P. aeruginosa* biofilm formation and reduce the expression of QS regulatory genes and QS-regulated genes [10]. Because *P. aeruginosa* causes an acute infection in the zebrafish embryo model, the reduced embryo mortality observed upon C11 addition in vivo is likely due to its ability to downregulate QS gene expression, which in turn impairs virulence, rather than a proper anti-biofilm effect. In addition or alternatively, we cannot exclude that C11 may also promote immunomodulatory mechanisms in vivo and stimulate a protective innate immune response.

To summarize, we have validated the use of static immersion of injured embryos for the assessment of *P. aeruginosa* virulence and the evaluation of anti-*Pseudomonas* molecules for the first time. The main advantage of this method is its rapidity and feasibility, making it suitable for the in vivo screening of novel anti-*P. aeruginosa* molecules. This method, which has confirmed the therapeutic potential of the C11 anti-QS compound, should thus be suitable to validate in vivo other molecules targeting QS, as well as molecules targeting other virulence factors important for *P. aeruginosa* pathogenesis in zebrafish embryos. In the current model, *P. aeruginosa* causes an acute infection, and it would be of importance to identify conditions for a more persistent infection to also assess the efficiency of QS inhibitors in such conditions.

## 4. Materials and Methods

### 4.1. Bacterial Strains and Growth Conditions

We used *P. aeruginosa* PAO1 strain or PAO1 (H103) constitutively expressing green fluorescence protein (GFP*mut2*) [38] to visualize bacteria in zebrafish embryos. GFP-expressing *oprF* mutant (H636) was described previously [32]. Bacteria were grown at 37 °C in Luria broth (LB). Carbenicillin (150 μg/mL) was added for strains expressing GFP. Bacterial growth was monitored in 96-well plates using a Spark 20M (Tecan, Trading AG, Männedorf, Switzerland) microplate reader.

### 4.2. Chemicals

*N*-(2-Pyrimidyl)butanamide (MW 165.19), named C11, was synthesized as described [10,39] and dissolved at 19 mM in DMSO or H_2_O. For bath infection, the stock solution was first diluted to 1.9 mM by adding phosphate-buffered saline (PBS) drop by drop and then diluted at the indicated concentration (10, 25, or 50 µM). 

Ciprofloxacin (Sigma-Aldrich, St-Louis, MO, USA, MW 331.35) was dissolved at 25 mg/mL in 0.1 M hydrochloric acid.

### 4.3. Bath Infection of Danio rerio Embryos with P. aeruginosa

Bacterial strains, freshly streaked out from glycerol stock, were grown overnight at 37 °C in LB medium (with antibiotic when required). Cultures were diluted 1:20 in LB medium and grown until OD_600_ = 0.9. Bacteria were centrifuged at 4000 rpm for 10 min and resuspended at the desired concentration in fish water composed of 60 µg/mL sea salt (Instant Ocean, Blacksburg, VA, USA) in distilled water with 4.10^−4^ N NaOH or N-phenylthiourea (PTU) (for experiments with microscopic analysis). The number of bacteria in the inoculum was determined by subsequent plating onto LB agar after dilution into PBS.

Experiments were performed using the AB zebrafish line or the GAB zebrafish line and maintained at 28 °C under standard conditions in fish water [25]. Bath immersion infections were done at 48 hpf on embryos dechorionated at 30 hpf. To injure the tail fin before infection, embryos were placed in a Petri dish and anesthetized with 40 µg/mL tricaine, and a small transection of the tail was performed with a 26-gauge needle under a stereomicroscope (Motic, Barcelona, Spain). 

For initial experiments on healthy embryos, groups of 20 larvae were distributed into 6-well plates (Falcon) containing 4.0 mL of fish water or bacterial suspension. For experiments on injured embryos, groups of 20 injured embryos were first immersed in a Petri dish containing bacterial suspension (or fish water as control) immediately after the transection of the tail and were then distributed into 96-well plates (Falcon^TM^, Glendale, AZ, USA) with two fish per well containing 200 µL of bacteria suspension (or fish water). The plates were incubated at 28 °C (bacteria are kept throughout the experiment in fish water, which does not support *P. aeruginosa* growth). For survival kinetics after infection, the number of dead embryos was determined visually based on the absence of heartbeat.

### 4.4. Fluorescence Microscopic Analysis of Zebrafish Embryos

Embryos were maintained in fish water containing PTU 1X added at 24 hpf to prevent pigment formation. Groups of five injured embryos were distributed into 6-well plates containing 4.0 mL of GFP-expressing bacteria suspension in PTU. After 1.5 or 24 hours of bath immersion, embryos were washed four times in PTU and kept for 50 min in PTU 1X. For live imaging, embryos from bath infection were placed in a glass-bottom Petri dish for inverted microscopy containing tricaine (250 µg/mL) in PTU. Direct visualization was performed using an Olympus MVX10 epifluorescent microscope equipped with a digital color camera (Olympus XC50). Fluorescence and bright-field images were acquired and processed with CellSens (Olympus) with GFP filter and assembled using ImageJ software to adjust levels and brightness of fluorescence.

### 4.5. Infection of Danio rerio Embryos by Microinjection in the Caudal Vein

Experiments were performed using the AB zebrafish and maintained under standard conditions [25]. Bacterial strains, which were freshly streaked out from glycerol stocks, were grown in LB medium to mid-log phase (OD_600_ = 0.7–0.8), recovered by centrifugation, and washed twice in PBS. Suspensions were homogenized through a 26-gauge needle and resuspended in PBS at about 10^9^ bacteria/mL added with 10% phenol red to aid visualization of the injection process. Infection was carried by the direct microinjection of 2 nL of bacterial suspensions into the caudal vein of 50 hpf embryos, previously dechorionated and anesthetized with 0.02% tricaine. For survival kinetics after infection, the number of dead embryos was determined visually based on the absence of heartbeat.

### 4.6. Treatment of Bath Infected Embryos with Ciprofloxacin or C11

All experiments were conducted on groups of 20 injured embryos in 96-well plates (two embryos per well) containing 200 µL of fish water supplemented with bacterial suspension and molecule or solvent control. Toxicity was addressed with embryos treated only with the molecule but not bacteria.

Ciprofloxacin was added after 2 hours of incubation to each well containing injured embryos to reach a final concentration of 50 or 1 µg/mL. Negative control was done with HCl (0.2 and 0.5 mM, respectively). 

Bacterial suspension was added drop by drop to C11 to reach a final concentration of 10, 25, or 50 µM before immersion of injured embryos. For non-treated controls, bacterial suspension was added to adjusted volumes of DMSO to reflect the DMSO amount present in treated samples.

### 4.7. Statistical Analysis

All statistical analyses were performed using GraphPad Prism 5 (Graphpad Software, San Diego, CA, USA). To compare the distribution of two groups, the Kruskal–Wallis test was performed. Comparisons between survival curves were performed using the log-rank test. In figures, * means *p*-value < 0.05, ** < 0.01, and *** < 0.001.

### 4.8. Ethics Statement

All animal experiments described in the present study were conducted at the University of Montpellier according to European Union guidelines for handling of laboratory animals (http://ec.europa.eu/environment/chemicals/lab_animals/home_en.htm (accessed on 15 March 2021)) and were approved by the Direction Sanitaire et Vétérinaire de l’Hérault and Comité d’Ethique pour l’Expérimentation Animale under reference CEEA-LR- B4-172-37 and APAFIS#5737-2016061511212601 v3. The breeding of adult fish adhered to the international guidelines specified by the EU Animal Protection Directive 2010/63/EU, and adult zebrafish were not sacrificed for this study. All experiments were performed before the embryos’ free-feeding stage and did not fall under animal experimentation law according to the EU Animal Protection Directive 2010/63/EU. For survival curves, cardiac rhythm was used as a clinical criterium. Embryos were euthanized using the anesthetic tricaine up to a lethal dose (500 mg/mL) before bleach treatment.

## Supplementary Materials

The following are available online at https://www.mdpi.com/article/10.3390/pathogens10040401/s1, Figure S1: Immersion of healthy embryos with *P. aeruginosa*. Figure S2: Efficiency and toxicity of C11 molecule dissolved in water.
