# Supplementary material for: A Novel Infection Protocol in Zebrafish Embryo to Assess Pseudomonas aeruginosa Virulence and Validate Efficacy of a Quorum Sensing Inhibitor In Vivo
Source: Pathogens. 2021 Mar 29;10(4):401. doi: 10.3390/pathogens10040401 (PMC8065929; doi:10.3390/pathogens10040401)
Supplement: Supplementary file 1 [file pathogens-10-00401-s001.pdf]

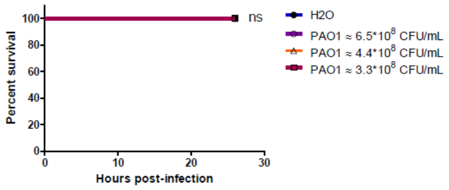

Fig. S1: Immersion of healthy embryos with *P. aeruginosa*. Survival curves (Kaplan-Meier representation) of healthy embryos (AB line, n=20 larvae per condition) immersed at 48 hpf with PAO1 wild-type strain grown in stationary phase or “fish-water”(negative control). No difference was found (p-value > 0,05).

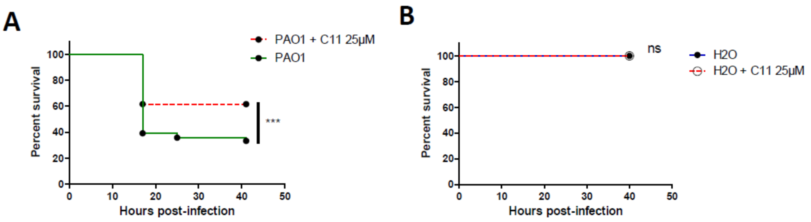

Fig. S2. Efficiency and toxicity of C11 molecule dissolved in water. (A) The antivirulence efficacy of C11 was tested with embryos injured in the tail fin and bath infected with PAO1 suspension at approximately $7*10^7$ CFU/mL in presence of C11 dissolved in H_2O at 25 µM. A significant difference (****P*<0.001) is found in the survival curve of C11 treated embryos comparatively to non treated embryos. **(B)** The toxicity of C11 at 25 µM (dissolved in H_2O) was monitored after immersion of embryos injured in the tail fin. For all experiments, the embryo survival was monitored for 45 hours and survival curves were represented with a Kaplan-Meier representation. Graphs represent the pool of three independent experiments (n = 60 larvae in total per condition).
